# Interneuron and glial mechanisms underlying V1 orientation map dynamics

**DOI:** 10.1016/j.ibneur.2026.01.017

**Published:** 2026-02-02

**Authors:** Lewen Zhao, Xingyuan Liu, De-hua Wu, Wei-Qun Fang

**Affiliations:** a Department of Anesthesiology, Songjiang Research Institute, Shanghai Key Laboratory of Emotions and Affective Disorders, Songjiang Hospital Affiliated to Shanghai Jiao Tong University School of Medicine, Shanghai 201600 China; b Department of Anatomy and Physiology, Shanghai Jiao Tong University School of Medicine, Shanghai 200025 China; c Department of Anesthesiology, Songjiang Hospital Affiliated to Shanghai Jiao Tong University School of Medicine, China

**Keywords:** Neurodevelopment, Visual Cortex, Orientation Selectivity Map, Orientation Column, Neuron-glial Interaction, Experience-dependent plasticity

## Abstract

The functional architecture of the primary visual cortex (V1)—manifesting as orientation selectivity maps (OS maps) in higher mammals and modular tuning clusters in rodents—provides a window into the rules governing cortical circuit organization. Although OS maps emerge from intrinsic activity and the structured sampling of retinothalamic inputs, their maturation and lifelong adaptability depend on cellular mechanisms that extend far beyond excitatory wiring. Recent advances indicate that inhibitory interneurons and glial cells play critical modulatory roles in map assembly, stabilization, and experience-dependent plasticity. Inhibitory interneurons regulate excitatory–inhibitory (E/I) balance, stabilize population responses, and gate developmental and adult plasticity through coordinated local and long-range interactions. In parallel, astrocytes modulate circuit excitability and experience-dependent refinement by regulating synaptic signaling, metabolic and ionic homeostasis, and plasticity-related receptor function, whereas microglia influence map formation and maintenance indirectly through activity-dependent synaptic remodeling and network homeostasis. This minireview synthesizes emerging insights into how inhibitory networks and glial–neuron crosstalk jointly orchestrate the formation and experience-dependent remodeling of orientation maps, offering a cellular framework for understanding the construction and flexibility of cortical sensory representations.

## 1 Comparative architecture: orientation selectivity maps across mammalian species

### 1.1 From columns to continuous maps

**Fig. 1:**
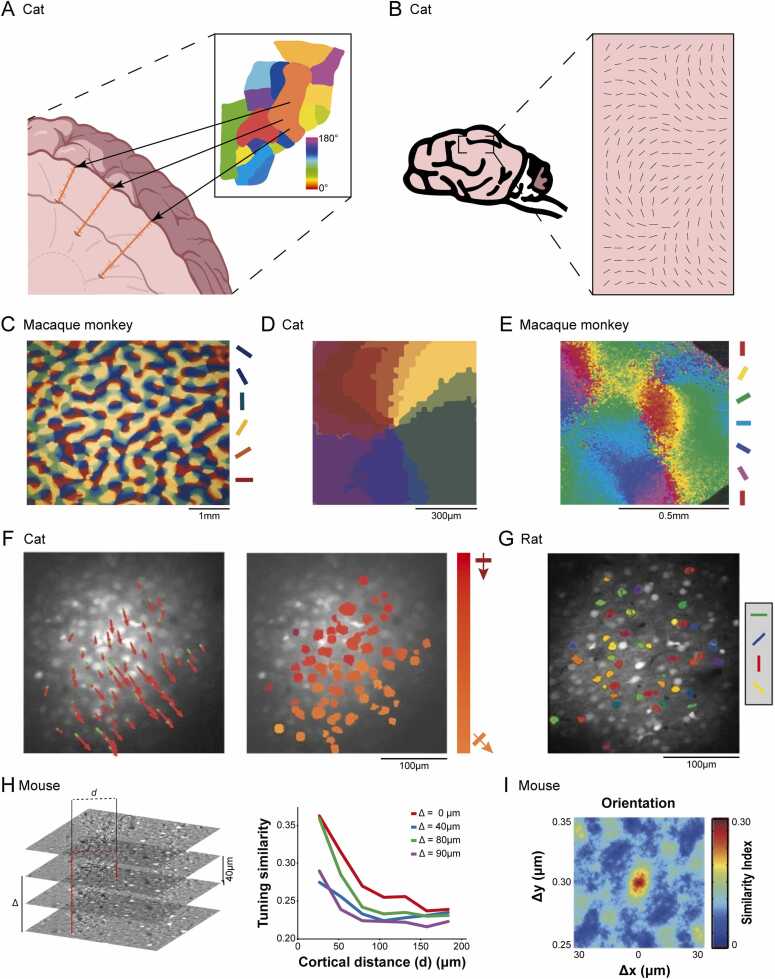
**Diverse Topographic Patterns of Neuronal Orientation Preference in Mammalian Visual Cortices.** (A) Neurons in the cat sensory cortices exhibit distinct and segregated orientation preferences during vertical electrode penetrations in response to stimulation at different peripheral receptive fields. (B) With denser electrode recordings, a transitional pattern of orientation preference across the cat visual cortex becomes evident. (C) Patterns of orientation selectivity were mapped to the monkey striate cortex using voltage-sensitive dye coupled with in vivo optical imaging. Patches of the same color represent iso-orientation activity patterns evoked by their preferred orientation. (D) A close-up of orientation center. Cat visual cortex exhibits highly ordered pinwheel-like patches of iso-orientation domains, where orientation preference of neurons changes continuously around the center, rather than elongated stripe-like structures described in (A). (E) Orientation preference maps of macaque monkey, measured using large-scale single-cell resolution two-photon calcium imaging. (F) The left panel displays a continuous vector map of preferred directions in cat visual cortex. Red and green vectors denote preferred and null direction responses, respectively. Preferred directions of identified cells vary smoothly and orderly (gradually shifting down-right), with each neuron responds similarly to its nearest neighbours. The right panel shows the color-coded direction-preference map derived from the left panel. (G) Orientation functional map of rat primary visual cortex. Visually responsive cells are color-coded by their optimal orientation. Unlike the cat visual cortex, which exhibits discernible local structure, the rat primary visual cortex displays a salt-and-pepper pattern, where neighboring neurons often respond to different orientations. (H) In the mouse visual cortex, average tuning similarity of neurons decreases as a function of cortical distance, revealing a weak spatial clustering. (I) Orientation tuning similarity map suggests that in mouse primary visual cortex neurons with similar response selectivity are clustered horizontally in the scale of a minicolumn.

The idea of functional organization in sensory cortex emerged from mid-20th-century electrophysiology. Mountcastle’s seminal work in cat somatosensory cortex introduced the columnar hypothesis, proposing vertically aligned neurons with shared receptive-field properties as an elementary cortical unit (Mountcastle, 1957). Extending this concept, Hubel and Wiesel showed that vertical penetrations through cat visual cortex yielded neurons with similar orientation preferences, leading to the classic model of orientation columns (Hubel and Wiesel, 1962, Hubel and Wiesel, 1963) (Fig. 1A). Later studies using denser electrode sampling revealed that orientation preference changed gradually across the cortical surface, challenging the strictly modular, discrete-column view (Albus, 1975) (Fig. 1B). The advent of optical imaging, which allowed direct visualization of complex spatial organization of orientation preferences, provided definitive evidence: orientation preferences vary smoothly across the cortical surface, forming pinwheel-like patterns that organize V1 neurons into iso-orientation domains (IODs) and pinwheel centers (PCs) (Blasdel and Salama, 1986, Bonhoeffer and Grinvald, 1991) (Fig. 1C and D).

(a) Panel adapted from Hubel and Wiesel (1963). (b) Panel adapted from Albus (1975). (c) Panel adapted from Blasdel and Salama (1986). (d) Panel adapted from Bonhoeffer and Grinvald (1991). (e) Panel adapted from Nauhaus et al. (2012). (f) Panel F and G adapted from Ohki et al. (2005). (g) Panel adapted from Ringach et al. (2016). (h) Panel adapted from Kondo et al. (2016).

**Fig. 2:**
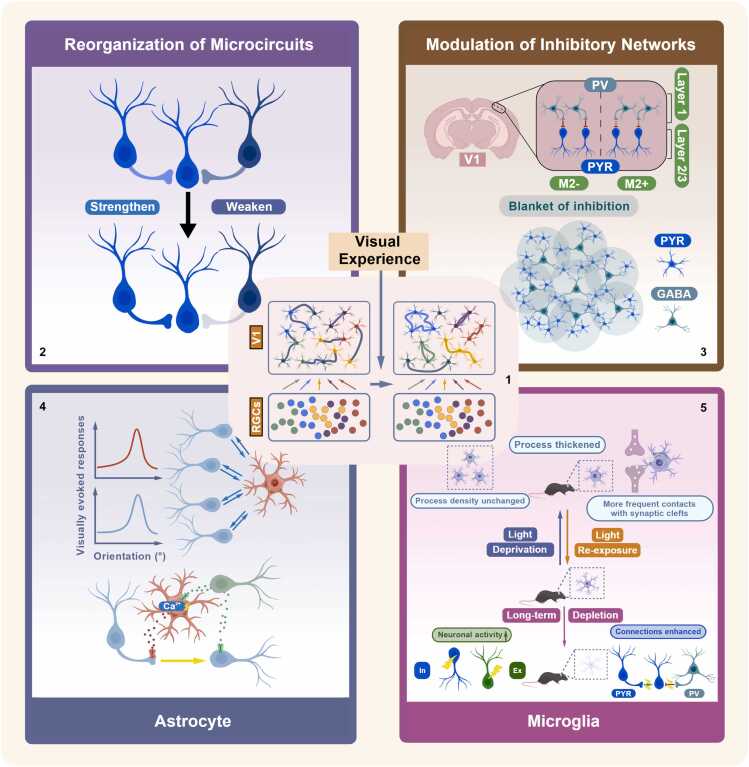
**Schematic Illustration of Factors Contributing to Functional Map Refinement and Maintenance.** (1) After the onset of visual experience, cortical circuits undergo substantial re-modeling where functional connections are selectively strengthened between neurons with similar visual preferences and pruned from visually unresponsive cells. Potential contributors of refinement and maintenance of local cortical network are further described in Panels 2–5. Synaptic connections are gradually strengthened between nearby neurons sharing similar functions, while connections between functionally different neurons are weakened, leading to the maturation of microcircuits. Maturation of inhibitory networks may play a critical role in formation of functional organization. Top, layer 1 inhibitory neurons of mouse V1 contains modular subregions distinguished by differential expression of M2 muscarinic receptors and exerts differential inhibitory control over downstream pyramidal neurons in layer 2/3. Bottom, GABAergic interneurons innervate nearby pyramidal neurons in a dense and non-specific way, forming a “blanket of inhibition”. PV, PV interneurons; PYR, pyramidal neurons; GABA, GABAergic interneurons. Top, astrocytes not only integrate orientation preference of adjacent neurons, but also modulate visual responses of neighboring neurons. Bottom, astrocytes modulate synaptic plasticity and E/I balance through neurotransmitter integration and ionic buffering. Microglia actively participate in modification of synapses. Top, in juvenile mice, abnormal visual experience alters the morphology and location of microglia. Bottom, long-term microglial depletion increases excitatory and inhibitory activity, and enhances both local excitatory and inhibitory connections to excitatory neurons. In, inhibitory neuron; Ex, excitatory neuron. Created with BioGDP.com (Jiang et al., 2025).

### 1.2 Species diversity and shared local logic

This pinwheel-based architecture is well established in higher mammals including primates (Blasdel and Salama, 1986, Nauhaus et al., 2012, Wiesel and Hubel, 1974) (Fig. 1E), cats (Bonhoeffer and Grinvald, 1991, Ohki et al., 2006), tree shrews (Bosking et al., 1997) and ferrets (Chapman et al., 1996, White et al., 2001). Rodents, by contrast, lack large-scale modularity and display a salt-and-pepper organization in which orientation-selective neurons are intermixed without global order (Bonin et al., 2011, Girman et al., 1999, Ohki et al., 2005, Van Hooser et al., 2005) (Fig. 1G).

Nonetheless, recent high-resolution recordings reveal consistent microscale clustering in rodent V1, where tuning similarity decreases systematically with distance (Kondo et al., 2016, Ringach et al., 2016) (Fig. 1H and I). Across species, a common principle persists: nearby neurons tend to share similar orientation preferences, whether arranged into smooth maps or dispersed clusters. This conserved local organization provides the structural substrate upon which experience-dependent refinement and plasticity operate.

## 2 Theoretical models of orientation selectivity maps formation

### 2.1 Retino-cortical sampling as a predictor of map formation

A longstanding theoretical question is why certain mammals develop large-scale, continuous OS maps. Early frameworks proposed that map emergence depends on sampling density across the retino–thalamic–cortical pathway. Species with high central-to-peripheral (CP) retinal density ratios, which indicates a dense central sampling to attain high visual acuity, were predicted to develop orientation columns (Ibbotson & Jung, 2020). Although direct link between CP ratio and cortical map formation is lacking, high CP ratios are associated with a predominance of retinal inputs routed through the LGN and an expanded V1 representation which is characteristic of primates and carnivores. This concept was expanded by the retino-cortical (RC) ratio theory, which proposes that OS maps appear when V1 receives sufficiently dense retinocortical input to capture Moiré interference patterns in ON-OFF retinal mosaics (Jang et al., 2020). Another complementary theory suggests that OS maps emerge through cortical sorting guided by the established “like-to-like” rules of thalamocortical convergence (Alonso et al., 2001, Clay Reid and Alonso, 1995). When thalamic afferent density is high, cortical circuits can not only faithfully sample these structured input templates but also refine them through the segregation of thalamic inputs along multiple stimulus dimensions including orientation preference (Najafian et al., 2022). These models collectively indicate that OS maps arise from the interplay between inherited anisotropies in retinal sampling and cortical self-organization mechanisms.

### 2.2 Computational significance of the pinwheel architecture

The pinwheel architecture characteristic of map-bearing species conveys important computational advantages. Iso-orientation domains consist of high-contrast, sharply tuned neurons characterized by robust cross-orientation suppression, optimized for encoding precise edge orientations. In contrast, pinwheel centers exhibit broader tuning and integrate multiple orientation components, making them well suited for detecting complex textures and patterns (Goris et al., 2015, Koch et al., 2016, Nauhaus et al., 2008). This functional dichotomy supports efficient coding of natural scenes by distributing different computations across spatially organized subdomains. Recent imaging and computational work further suggests smoother tuning gradients rather than discrete compartments (Li et al., 2019) and a temporal hierarchy: PCs may act as rapid saliency detectors that initiate spiking waves propagating to adjacent IODs, which then refine edge representations for clear perception (Zhong et al., 2024). Together, these studies depict the pinwheel map as a sophisticated computational architecture that leverages spatial and temporal specialization to extract the rich statistics of natural scenes.

### 2.3 Shared organizing principles across species

Even in rodents—where OS maps do not form at the macroscopic scale—the underlying circuit organizing rules remain highly conserved. High-resolution imaging reveals that similarly tuned neurons exhibit consistent microscale clustering. At the synaptic level, excitatory neurons preferentially form connections with functionally similar partners (Ding et al., 2025, Ko et al., 2011), indicating that fine-scale subnetworks in rodents may be structurally and functionally analogous to the iso-orientation domains in map-bearing species. Nonetheless, general theoretical frameworks, such as RC ratio and thalamic afferent sampling density, successfully predict map formation features across species, suggesting common developmental constraints. More importantly, the key mechanisms that shape orientation tuning and its plasticity, including E/I balance, rules of feedforward and recurrent wiring, activity-dependent synaptic modification, and glia-dependent remodeling, are preserved across mammalian cortices. Thus, despite architectural differences, theoretical models and conserved cellular principles together provide a unified framework for studying how OS maps form, stabilize, and adapt to experience.

## 3 Intrinsic drivers: the role of spontaneous activity

The maturation of V1 circuits in mammals involves a synergistic interplay between intrinsic genetic programs and spontaneous neuronal activity. Before the onset of vision, molecular cues and patterned spontaneous activity cooperate to organize the early cortical proto-map (Feldheim and O′Leary, 2010, Huberman et al., 2008). This pre-experience phase constructs a functional scaffold, which is later calibrated and sharpened by visual experience.

### 3.1 Retinal waves as structured teaching signals

Retinal waves—spontaneous bursts of action potentials that propagate across neighboring retinal ganglion cells (Meister et al., 1991)—are capable of coordinating patterned activity across the developing visual system (Ackman et al., 2012). These patterned waves propagate to the LGN, driving correlated activity and synaptic remodeling that is essential for refining retinothalamic (Butts et al., 2007, Feller, 2009, Mooney et al., 1996) and subsequent thalamocortical connections. Disruption of these waves leads to imprecise anatomical and functional mapping from the LGN to the visual cortex (Cang et al., 2005). Remarkably, when retinal projections are experimentally rerouted to the auditory pathway, orientation modules resembling those in V1 can also be induced in the primary auditory cortex (Sharma et al., 2000), demonstrating that the statistics of spontaneous input itself could determine the early cortical scaffold.

### 3.2 Self-organization and early network dynamics in cortex

Modular patterns of both spontaneous and stimulus-evoked activity (measured with eyelids manually separated) emerge in visual cortex well before natural eye opening (Chapman et al., 1996, Crair et al., 1998, White et al., 2001). Spontaneous activity patterns in naive ferret V1 before eye opening can partially predict the mature OS map (Smith et al., 2018). Once established, these immature cortical networks can reorganize unstructured inputs into spatially periodic activity patterns with a characteristic spacing (Mulholland et al., 2024), and are resilient to external perturbations such as temporary silencing of feedforward inputs from the retina or LGN (Smith et al., 2018), exhibiting a remarkable capacity for self-organization. Together, spontaneous retinal waves and intrinsic cortical dynamics establish a proto-map that anticipates the mature orientation architecture. This innate scaffold is then refined by visual experience through inhibitory networks, glial modulation and ongoing plasticity.

## 4 Plasticity and remodeling: experience-dependent modifications

Following eye opening, V1 circuits transition from intrinsically patterned scaffolds to finely tuned networks capable of reliably encoding visual features. While dark-reared animals retain rudimentary orientation maps, these maps are weakly tuned, and degraded visual input further compromises map precision (White et al., 2001). At the onset of vision, evoked activity patterns are highly variable and poorly discriminate between different stimuli, but later stabilize and converge with spontaneous activity patterns, indicating the reorganization of both feedforward inputs and local cortical circuits (Tragenap et al., 2025). Collectively, these observations highlight that visual experience provides instructive signals essential for the fine-scale refinement of functional maps.

### 4.1 Experience shapes representational space

Classic stripe-rearing experiments provided compelling evidence that experience can reshape cortical topography. Kittens exposed to a single orientation develop maps dominated by neurons tuned to that orientation, with non-experienced orientations severely underrepresented (Blakemore & Cooper, 1970). Optical imaging revealed that experienced orientations can expand their cortical territory up to two-fold as to the orthogonal one (Sengpiel et al., 1999), demonstrating that representational resources are highly plastic and dynamically allocated toward dominant environmental features.

### 4.2 Intrinsic bias versus experience-driven refinement

This form of experience-dependent plasticity intersects with the long-recognized oblique effect (Appelle, 1972): humans and many other species exhibit superior acuity for cardinal (horizontal and vertical) over oblique orientations. This perceptual bias appear partly intrinsic, where optical imaging in the visual cortex of monkeys (Xu et al., 2006), cats (Wang et al., 2003), and ferrets (Chapman and Bonhoeffer, 1998, Coppola et al., 1998) consistently reveals an over-representation of cardinal contours. Given the statistical prevalence of cardinal edges in natural scenes (Coppola, Purves, et al., 1998), it is reasonable to infer that this anisotropy might be shaped by biased experience. However, the fact that anisotropy in juvenile ferrets decreases after eye opening, and that dark-reared animals develop an even more pronounced anisotropy than visually exposed animals (Coppola & White, 2004) suggests that visual experience does not generate anisotropy de novo but refines and normalizes the intrinsic bias instead.

### 4.3 Refinement at cortical network level

Experience-dependent refinement relies on selective synaptic remodeling. Key contributors likely include the reorganization of local microcircuits—co-active, similarly tuned neurons strengthen their connections while uncorrelated or inappropriate synapses are pruned (Ko et al., 2013)—and the maturation of long-range horizontal connections (Durack and Katz, 1996, Ruthazer and Stryker, 1996), both of which enhance the stability and structure of evoked activity patterns. Suppression of cortical activity (Hagihara et al., 2015) or dark-rearing (Ko et al., 2014) disrupt the reorganization of network-level connectivity while leaving single-neuron orientation selectivity largely intact (Sherk & Stryker, 1976), highlighting that plasticity operates primarily at the population and network level.

Together, visual experience sculpts the intrinsically generated map into its adult form, reallocating representational space and refining synaptic connectivity. This interplay between intrinsic scaffolds and activity-dependent remodeling emphasizes the decisive role of experience in shaping mature orientation selectivity maps.

## 5 Inhibitory circuits and orientation selectivity map

### 5.1 Blanket of inhibition scales map expression

Although excitatory inputs provide the initial spatial template for the map, intracortical γ-aminobutyric acid (GABA)ergic inhibition is indispensable for translating this template into a stable and interpretable functional architecture. Pharmacological manipulations in cat V1 provide particularly compelling evidence. Global GABAA receptor blockade induces cortical epilepsy and entirely abolishes the orientation map, yet partial suppression of excitation restores a weakened map pattern (Yu et al., 2008). Structurally, most subtypes of GABAergic interneurons have extensive connectivity with excitatory neighbors (Karnani et al., 2014), and are often synchronized via gap junctions (Gibson et al., 1999), forming a dense and unspecific “blanket of inhibition” on local pyramidal neurons. Theoretical models suggest that such dense, lateral inhibition enables a “winner-takes-all” strategy that suppresses weaker neighboring activity, thereby enhancing the discrimination among similar sensory inputs. Such competitive dynamics are proposed as the driving force for the formation of self-organizing maps (Kohonen, 2006).

### 5.2 Inhibition develops in parallel with map refinement and gates plasticity

Recent evidence from ferret V1 suggests that this inhibitory control is not merely a late-emerging, non-specific blanket of activity, but is instead rooted in early-established, functionally specific networks. Even before the onset of visual experience, GABAergic interneurons in ferret V1 exhibit modular patterns of spontaneous activity that are tightly coupled—both globally and locally—with excitatory networks, showing quantitative agreement in their spatial frequency and correlation structure (Mulholland et al., 2021). The presence of sharp fractures of correlation in these early inhibitory networks argues against the traditional view of non-specific local pooling, suggesting that inhibitory neurons are integrated into specific functional scaffolds from the beginning.

In ferret V1, GABAergic interneurons acquire modular response patterns and robust orientation tuning only after eye opening, in parallel with the refinement of the cortical map. While the structural transition from wide-spread synchronization to modularity can proceed without visual experience, the reliable association of these patterns to oriented stimuli and maintenance of short-range correlations critically requires visual experience (Chang & Fitzpatrick, 2022), indicating inhibition’s role in experience-dependent map maturation. Furthermore, inhibitory tone also governs adult plasticity. In adult cats, transient enhancement of cortical excitability using high-frequency transcranial magnetic stimulation (TMS) creates a permissive window, during which visual training with a single orientation expands its cortical representation (Kozyrev et al., 2018). Thus, inhibitory circuits not only shape the moment-to-moment expression of OS maps but also affect when, and how strongly, these maps can be remodeled across life.

### 5.3 Modular inhibitory circuitry in rodent cortex

While inhibitory connectivity forms a dense blanket ensuring broad coverage, recent studies in mouse V1 have further shown that the strength of this inhibition is spatially modulated by long-range inputs arriving in cortical layer 1. Layer 1 is organized into patches enriched for M2 muscarinic receptor-expressing (M2^+^) neurons and complementary M2^-^ interpatches, forming subnetworks with distinct E/I dynamics and long-range connectivity (D'Souza et al., 2019, Ji et al., 2015). M2^+^ patches receive clustered thalamic and top-down input, whereas M2^-^ interpatches are targeted by higher-order thalamic nuclei and house parvalbumin-expressing (PV) interneurons that deliver stronger, faster inhibition to L2/3 pyramidal cells. This architecture produces functional specialization where M2^+^ neurons exhibit high spatial acuity and M2^–^ neurons show high temporal acuity, revealing that even salt-and-pepper cortex maintains structured heterogeneity in inhibitory control.

Together, these findings position the inhibitory network as a foundational organizer of OS maps: it stabilizes spatial patterns generated by excitation, introduces functional specialization, and enables map reorganization in adulthood. Yet the execution and maintenance of this inhibitory choreography rely on an active non-neuronal environment, setting the stage for the role of astrocyte, microglia, and extracellular matrix in sculpting map plasticity.

## 6 Beyond the neuron: glial mechanisms in map assembly and refinement

### 6.1 Astrocytic networks: integrators, modulators, and activity shapers

Glial cells are increasingly recognized not merely as passive support, but as active participants in shaping cortical functional architecture. Astrocytes tightly track local neuronal dynamics: in V1 they display sharp orientation selectivity that closely matches the preferences of adjacent neurons (Schummers et al., 2008, Slezak et al., 2019). This tuning reflects not only integration but also modulation, as optogenetic activation of V1 astrocytes alters the orientation selectivity of nearby neurons (Perea et al., 2014), while cholinergic-mediated activation of astrocytes induces stimulus-specific potentiation of visual responses, directly linking them to the mechanisms of map plasticity (Chen et al., 2012).

Beyond these direct modulatory effects, astrocytes also indirectly regulate the fundamental substrates underlying OS map formation and maintenance: experience-dependent refinement and circuit excitability. Astrocytes act as one of the gates for experience-dependent refinement. For instance, by regulating extracellular levels of co-agonists like D-serine, they could modulate N-methyl-D-aspartate (NMDA) receptor tone, thereby influencing the long-term synaptic changes (Henneberger et al., 2010, Panatier et al., 2006). Meanwhile, astrocytic glutamate modulation (Verhoog et al., 2020) and K⁺ clearance mechanisms (Bellot-Saez et al., 2017) constrain neuronal excitability, which further stabilize E/I balance that determines whether OS maps can be functionally expressed.

Astrocyte precursors (radial glia) also contribute to the basic scaffolding of circuits by providing physical and molecular cues for axon pathfinding during early development (Rapti, 2023). This is observed in the mouse optic chiasm (Mason & Sretavan, 1997), where radial glia demarcate RGCs axon paths and prevent inappropriate crossing or misrouting, although direct evidence for their role in guiding thalamocortical or long-range horizontal connections within V1 is still limited.

Astroglial spatial organization may also contribute to the geometry of OS maps. In ferret V1, astrocytes are typically arranged in a tiled pattern, sharing ∼50 % territory overlap with neighboring astrocytes (López-Hidalgo et al., 2016), suggesting a differential regulation on neuronal activity within overlapped regions. Modeling studies predict that altering astrocytic domain size or coupling strength can reshape map periodicity and modular boundaries (Philips et al., 2017). Evidence from other modular cortices (e.g., barrel cortex and olfactory glomeruli), where astrocyte networks are restricted and align with functional compartments (Houades et al., 2008, Roux et al., 2011), suggests a potentially common principle. In visual pathway, astrocytes in mice superior colliculus form extensive networks in the retinorecipient layer and are essential for functional orientation preference maps, but these networks are much sparser and their regulation does not occur in V1 (Visser et al., 2024). Whether such modular astrocytic lattices exist in columnar species and whether they position or stabilize OS modules remains an unresolved but compelling direction.

### 6.2 Microglia: synaptic sculptors with unresolved specificity

Microglia, the resident immune cells of the brain, play critical roles in synaptic pruning and neuronal activity homeostasis. Typically during development of visual pathway, microglia participate in retinogeniculate synaptic pruning which is dependent upon neural activity and the microglia-specific phagocytic signaling pathway (Schafer et al., 2012, Stevens et al., 2007; but see O’Keeffe et al., 2025). In juvenile mouse visual cortex, visual deprivation alters microglial morphology and motile states, and re-exposure to light can reverse these changes (Tremblay et al., 2010). However, the necessity of microglia for experience-dependent plasticity remains debated, as neither genetic deletion of the fractalkine receptor CX3CR1 nor pharmacological microglial depletion disrupts ocular dominance plasticity (Brown et al., 2024, Schecter et al., 2017). It is notable that while most existing studies focus on the consequences of microglial depletion, effects of microglial overactivation, observed in pathological conditions such as traumatic brain injury, post-traumatic stress disorder and depression, remain largely unexplored in the context of visual circuit development (Cornell et al., 2022).

From a homeostatic perspective, long-term microglial depletion in juvenile mice increases evoked excitatory and inhibitory activity measured in adulthood, which leads to reduced orientation selectivity (Velez et al., 2022). Consistently, chronic microglial depletion in adult mice enhances local excitatory and inhibitory connections to excitatory neurons and elevates the activity of both excitatory neurons and PV interneurons in visual cortex, indicating a disruption of network-level homeostasis (Liu et al., 2021). On the contrary, short-term microglial depletion during juvenile stages did not alter orientation tuning when assessed at P28-P32 (Brown et al., 2024). This discrepancy suggests that compensatory mechanisms operating during early development, such as homeostatic synaptic plasticity mediated by non-microglial cell types, may transiently preserve functional tuning, with deficits emerging only later in life (Turrigiano, 2012).

Together, these findings position neuron–glia interactions as potential contributors to OS map assembly and refinement. Astrocytes stabilize E/I balance and convert visual experience into plasticity signals, while microglia shape synaptic architecture through activity-dependent pruning. Yet despite growing evidence, the necessity and sufficiency of astrocytic gap-junction networks and microglial signaling pathways for establishing the large-scale spatial pattern of OS maps in higher mammals remain key open questions for the field.

## 7 Conclusion and future perspectives

OS maps emerge from the coordinated interplay of intrinsic spontaneous activity, structured retinocortical input, and experience-dependent refinement, all stabilized by a precisely tuned E/I balance. Early retinal-wave–driven activity provides a proto-map scaffold. Visual experience then progressively sharpens representational space through selective strengthening and pruning of synapses. Meanwhile inhibitory circuits scale and gate the functional expression of the map throughout life. Yet the assembly and remodeling of OS maps cannot be fully understood without incorporating the essential roles of glial networks. Astrocytes regulate local E/I homeostasis and transform sensory experience into modulatory signals that influence neuronal tuning, while microglia sculpt synaptic architecture in an activity-dependent manner, though their necessity for large-scale OS map organization remains debated.

These interactions raise key unresolved questions: how astrocytic gap-junction networks interface with emerging orientation modules; under what developmental or network states microglia are required for refining orientation selectivity; and how disruptions in E/I balance or glial signaling alter map topology and sensory encoding. Answering these questions will require new experimental frameworks—multiphoton and holographic imaging of neuron–glia assemblies, high-density electrophysiology to track distributed E/I dynamics, cell-type–specific perturbations of interneuron and glial subtypes, and unified computational models that integrate activity patterns with map geometry. Such approaches are essential not only for understanding normal map formation but also for revealing how OS map dysfunction contributes to neurodevelopmental and psychiatric conditions marked by abnormal synaptic plasticity, E/I imbalance and impaired sensory processing. Ultimately, advancing OS map research demands a shift from a neuron-centric view to a multi-systems framework in which inhibitory circuits and glial networks jointly orchestrate the emergence, refinement, and stability of cortical functional architecture.

## Funding

This work was supported by the Foundation for National Key R&D Program of China (grant no. 2021ZD0202900 to W.-Q.F.), the 10.13039/501100001809National Natural Science Foundation of China (grant no. 32271023 to W.-Q.F.), the Shanghai Pujiang Program (grant no. 22PJ1408900 to W.-Q.F.), the Key Discipline Project of Shanghai Municipal Health Commission (2024ZDXK0050), the Program for Professor of Special Appointment (Eastern Scholar) at Shanghai Institutions of Higher Learning (grant no. BJ1–6200–21–0008 to W.-Q.F.), and Shanghai Frontiers Science Center of Cellular Homeostasis and Human Diseases.

## CRediT authorship contribution statement

**Lewen Zhao:** Writing – review & editing, Writing – original draft. **De-hua Wu:** Writing – review & editing, Funding acquisition, Conceptualization. **Xingyuan Liu:** Writing – review & editing, Writing – original draft, Visualization. **Wei-Qun Fang:** Writing – review & editing, Writing – original draft, Supervision, Funding acquisition.

## Declaration of Competing Interest

The authors declare that they have no known competing financial interests or personal relationships that could have appeared to influence the work reported in this paper.
